# Construction of a nitrogen-doped carbon quantum dot-silver nanoparticle composite fluorescence sensing system for the highly selective detection of thiram in soil and pears

**DOI:** 10.1039/d6ra04161b

**Published:** 2026-07-15

**Authors:** Shangyu Hou, Rong Yang, Pute Yang, Xiaoqian Liu, Yu Gu, Qin Zhou

**Affiliations:** a College of Advanced Agriculture and Ecological Environment, Heilongjiang University Harbin 150080 China zhouqin@hlju.edu.cn; b Inspection and Test Center of Beet Quality, Ministry of Agriculture and Rural Afairs Harbin 150080 China

## Abstract

The widespread application of thiram poses potential threats to the ecological environment and human health. Existing detection methods rely on large-scale instruments and make it difficult to meet the requirements for rapid on-site detection. Therefore, in this study, nitrogen-doped carbon quantum dots (N-CQDs) were prepared by a one-step hydrothermal method using citric acid and melamine; the average particle size was 4.6 nm, and they exhibited good stability in aqueous solution. At the same time, silver nanoparticles (AgNPs) were synthesized by reducing nitric acid and sodium borohydride, with an average particle size of 7.6 nm. A fluorescence sensing system of N-CQDs/AgNPs was constructed based on the internal filter effect (IFE) for the highly selective detection of thiram. AgNPs can effectively quench the fluorescence of N-CQDs through the IFE; when thiram is present, the absorption spectrum of AgNPs shifts to the red, and the overlapping area between the fluorescence emission spectrum of N-CQDs and that of AgNPs increases, resulting in further fluorescence quenching. This system shows a good linear relationship in the concentration range of 0.007–5 µg mL^−1^ (*R*^2^ = 0.99298), and the detection limit is as low as 0.002 µg mL^−1^. This method is highly sensitive, selective, simple to operate, and capable of rapid detection, providing a new strategy for the on-site rapid detection of thiram residues in agricultural products and environmental samples.

## Abstract 2

Based on internal filter effect, N-CQDs/AgNPs sensor detects thiram. AgNPs quench N-CQDs; thiram red-shifts AgNP absorption, enhancing quenching. It is sensitive, selective, and rapid for on-site thiram analysis in agri/environmental samples.

## Graphical abstract

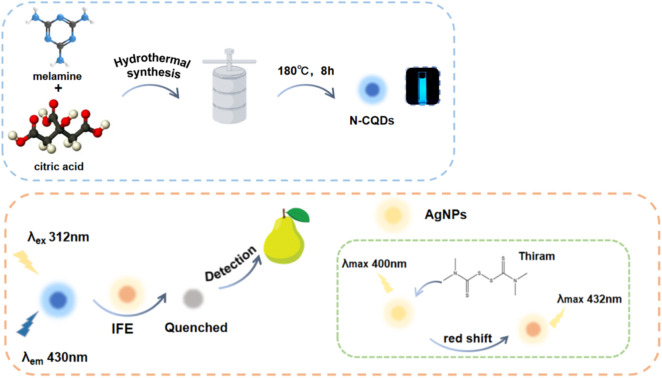

## 1. Introduction

Thiram belongs to the dithiocarbamate fungicide family and is characterized by the rapid onset of action, a long efficacy period, and low toxicity. It has been widely used to protect various agricultural products from fungal diseases.^1^ However, it exhibits strong irritant effects on the skin and mucous membranes, and long-term or excessive exposure may damage the nervous and digestive systems.^2^ Its residues in soil can affect the structure of soil microbial communities and disrupt the ecological balance. Currently, routine detection methods for thiram mainly include chromatographic methods (such as high-performance liquid chromatography^3^ and gas chromatography),^4^ as well as their hyphenated techniques with mass spectrometry,^5^ and enzyme-linked immunosorbent assays.^6^ Although these methods offer high sensitivity and accuracy, they typically rely on large-scale instruments, require complex sample pretreatment and necessitate skilled operators, making it difficult to meet the requirements for on-site rapid screening.^7^ Therefore, for the sake of environmental and food safety, it is crucial to develop accurate and convenient methods for thiram detection.

In recent years, fluorescence sensing technology based on nanomaterials has demonstrated immense potential in the field of pesticide residue analysis due to its advantages of simple operation, rapid response, low cost, and high sensitivity.^8^ To date, a large number of nanomaterials, such as nanomolecularly imprinted polymers,^9^ metal nanoparticles,^10^ and carbon-based nanomaterials,^11^ have been extensively developed due to their fascinating properties, and they are effectively applied in various fields. Carbon dots (CDs) are a recently developed class of nanomaterials within the carbon-based nanomaterial family; they are zero-dimensional photoluminescent nanocarbon, typically with a size of less than 10 nm.^12,13^ Generally, carbon dots (CDs) can be classified into several categories, including graphene quantum dots (GQDs), carbon quantum dots (CQDs), and carbon polymer dots (CPDs).^14^ Of these, carbon quantum dots, as an emerging fluorescent nanomaterial, have become ideal probes for constructing fluorescence sensors due to their excellent optical properties, low toxicity, and good biocompatibility.

Synthesis methods for CQDs mainly involve one of two strategies: top-down or bottom-up.^15^ Top-down strategies for synthesizing carbon quantum dots involve decomposing larger carbon structures, such as nanodiamonds, graphite, carbon nanotubes, carbon soot, activated carbon, and graphite oxide, through methods including arc discharge, laser ablation, and electrochemical oxidation. Bottom-up methods for synthesizing carbon quantum dots can be carried out through heat treatment, templating, electrochemical, and microwave synthesis routes,^16^ starting from molecular precursors (such as citrates, carbohydrates, *etc.*). Among the various synthesis methods, the use of small organic and biological molecules as carbon sources has become a sustainable and universal strategy.^17^ Carbon quantum dots (CQDs), as an excellent fluorescent nanomaterial, have found extensive applications in many fields due to their remarkable fluorescence characteristics, as well as outstanding optoelectronic properties, good water solubility, low toxicity,^18^ and superior biocompatibility.^19^ However, their relatively low quantum yields (QYs) remain an unignorable drawback, and doping carbon dots with heteroatoms can enhance the quantum yield.^20^ Nitrogen is one of the most common heteroatoms because it is very similar to carbon in terms of its electronic structure and size.^21,22^ Research by Zhan *et al.*^23^ shows that nitrogen doping is an effective strategy to improve the quantum yield, with the quantum yield increasing by 108.55% after doping compared to before. Ma *et al.*^24^ also increased the quantum yield of carbon quantum dots from 0.64% to 13.72% through nitrogen doping. Noble-metal nanoparticles are called excellent quenchers due to their high molar extinction coefficients in the visible light region and their tunable absorption spectra,^25,26^ and the properties of noble-metal nanoparticles have aroused great interest for optical sensing applications based on fluorescence quenching.^27^ At the same size, silver nanoparticles exhibit a much higher extinction coefficient than gold nanoparticles;^28^ therefore, silver nanoparticles are excellent candidates for fluorescence quenching applications based on the inner filter effect (IFE).^25^

In this study, nitrogen-doped carbon quantum dots (N-CQDs) were synthesized *via* a one-step hydrothermal method, and a fluorescence sensing system based on nitrogen-doped carbon quantum dots (N-CQDs) and silver nanoparticles (AgNPs) was constructed to achieve the rapid and highly sensitive detection of thiram.

## 2. Experimental section

### 2.1 Materials and instruments

A transmission electron microscope (JEM-2100, JEOL Corporation), Fourier-transform infrared spectrometer (Nicolet 6700, Thermo Fisher Scientific Inc.), X-ray photoelectron spectrometer (Escalab 250Xi, Thermo Fisher Scientific Inc.), X-ray diffractometer (Bruker AXSD 5005, Bruker Corporation), steady-state/transient fluorescence spectrometer (FLS1000, Edinburgh Company, UK), fluorescence spectrophotometer (A01-dz639-AR3-358, Varian Inc., USA), ultraviolet-visible spectrophotometer (UV-2450, Shimadzu Corporation), electric heating air oven (DHG-9055A, Shanghai Yiheng Scientific Instrument Co., Ltd), freeze dryer (TFD-1A-95+, Beijing Boymang Experimental Instrument Co., Ltd), and heat-purifying constant-temperature magnetic stirrer (DF-101S, Shanghai Xiluobai Leibo Instrument Co., Ltd) were used.

Citric acid (C_6_H_8_O_7_, AR ≥ 99%), melamine (C_3_H_6_N_6_, AR ≥ 99%), sodium citrate (C_6_H_5_Na_3_O_7_, AR ≥ 99%), sodium borohydride (NaBH_4_, AR ≥ 99%), silver nitrate (AgNO_3_, AR ≥ 99%), 3-(*N*-morpholino)propanesulfonic acid (MOPS, AR ≥ 99%), sodium bicarbonate (NaHCO_3_, AR ≥ 99%), sodium hydroxide (NaOH, AR ≥ 99%), nitric acid (HNO_3_, AR ≥ 99%), sodium chloride (NaCl, AR ≥ 99%), ferrous sulfate (FeSO_4_, AR ≥ 99%), copper sulfate (CuSO_4_, AR ≥ 99%), acetonitrile, and methanol were purchased from Shanghai Aladdin Biological Reagent Co., Ltd. Thiram, methyl parathion, acephate, and glyphosate were all purchased from the Environmental Quality Supervision, Inspection and Testing Center of the Ministry of Agriculture and Rural Affairs (Tianjin). All water used in the experiments was deionized water.

### 2.2 Synthesis of N-CQDs

8.4 g of citric acid and 5.04 g of melamine were dissolved in 80 mL of water, subjected to ultrasonic treatment for 10 minutes, and stirred until a uniform solution was obtained. Subsequently, the mixture was moved into a hydrothermal autoclave and heated in an oven at 180 °C for a duration of 8 hours. Once the reaction finished, the solution was left to cool down to room temperature naturally. The precipitate was removed by filtration, and the supernatant was filtered through a 0.22-µm membrane filter. The filtrate was then placed into a 1000-Da dialysis bag, dialyzed for 20 hours, and subsequently freeze-dried under vacuum for 24 hours to obtain N-CQD powder.^29^

### 2.3 Synthesis of AgNPs

250 µL of 0.1 mol per L AgNO_3_ solution, 0.1 mol per L Na_3_C_6_H_5_O_7_·2H_2_O solution, and 6 mL of 5 mmol per L NaBH_4_ solution were measured separately and then added dropwise into 90 mL of water under magnetic stirring. After the addition was complete, stirring was continued for another 30 minutes. The obtained product was allowed to stand in the dark for 8 h, then transferred to a 100-mL brown volumetric flask and made up to the mark with water. This was placed in a dark room at 4 °C and kept ready for use.^30^

### 2.4 The quantum yield of N-CQDs

The fluorescence quantum yield refers to the fraction of molecules in the excited state that return to the ground state by emitting fluorescence among all the excited-state molecules. The greater the quantum yield, the stronger the fluorescence of the quantum dots. Fluorescence quantum yield is generally measured by the reference method. In this experiment, quinine sulfate was selected as the reference substance, with a known quantum yield of 54%.^31^ The absorption spectra and the fluorescence spectra (with an excitation wavelength of 312 nm) of carbon quantum dot solution and quinine sulfate solution at appropriate concentrations were measured, respectively. In the formula for amplitude-weighted average lifetime (eqn (1)), *Q* represents the quantum yield, the subscript *R* in the fluorescence quantum yield indicates the reference substance quinine sulfate, *A* is the absorbance, and *n* is the refractive index of the solvent. As the solvents for both the carbon dot solution and the quinine sulfate solution are ultrapure water, *n*/*n*_R_ ≈ 1.1
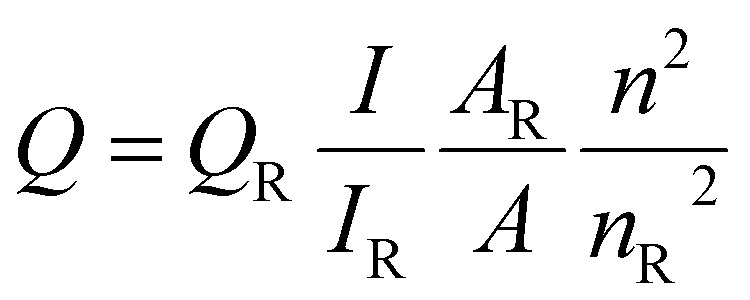


### 2.5 Detection of thiram

An appropriate amount of N-CQD powder was weighed and dissolved in water to prepare a 0.1 mg per mL N-CQD solution. The thiram standard was dissolved in methanol to prepare a 200 µg per mL stock solution, and it was diluted to the required concentrations with 10 mM MOPS buffer (pH = 6). 1 mL of AgNP solution was mixed with 1 mL of thiram standard solution at different concentrations (0–5 µg mL^−1^) in a 5-mL brown vial, then 1 mL of N-CQD solution was added. After shaking well, the reaction was allowed to proceed for 1 minute at room temperature. The fluorescence spectrum was measured at an excitation wavelength of 312 nm, and the fluorescence intensity was recorded at 430 nm. A standard curve was plotted by calculating the fluorescence change Δ*F* (Δ*F* = *F*_0_ − *F*, where *F*_0_ and *F* represent the fluorescence intensity before and after the addition of thiram, respectively) against the thiram concentration.

### 2.6 Selectivity experiments

Common interfering substances, such as pesticides and metal ions, were selected and added to the fluorescence sensing system. Fluorescence detection was performed under the same conditions, and the fluorescence spectra of the different reaction systems were recorded at an excitation wavelength of 312 nm.

### 2.7 Detection of thiram in real samples

Commercially available pears and soil samples were selected for real sample detection using the spiked recovery method. The specific pre-treatment procedures are as follows: 25.00 g of the sample was weighed, 50 mL of acetonitrile was added, and it was spiked with thiram standard solution to predetermined concentrations (0.5 µg mL^−1^, 1 µg mL^−1^, 3 µg mL^−1^, and 5 µg mL^−1^). The pear sample was homogenized, then filtered into a graduated cylinder with a stopper containing sodium chloride for salting out, and it was allowed to stand for 30 min. For the soil sample, an appropriate amount of diatomaceous earth was added, and it was placed on a shaker to oscillate for 30 min, let stand for 24 h, and then suction filtration was performed. 10 mL of the supernatant was taken from each of the two samples mentioned above, and rotary evaporation was performed. After evaporating to dryness and cooling to room temperature, the residue was re-dissolved with 5 mL of methanol, filtered through a 0.22-µm organic filter membrane, and stored at 4 °C. Fluorescence detection was carried out according to the method given in Section 2.5, and the recovery rate of the added substance was calculated.

## 3. Results and discussion

### 3.1 Characterization and stability of N-CQDs

By using techniques such as transmission electron microscopy (TEM), X-ray diffraction (XRD), Fourier-transform infrared spectroscopy (FTIR), X-ray photoelectron spectroscopy (XPS), ultraviolet-visible absorption spectroscopy (UV-vis), and fluorescence spectroscopy, the microscopic morphology, crystal structure, surface chemical state, and optical properties of the material were systematically characterized.

**Fig. 1:**
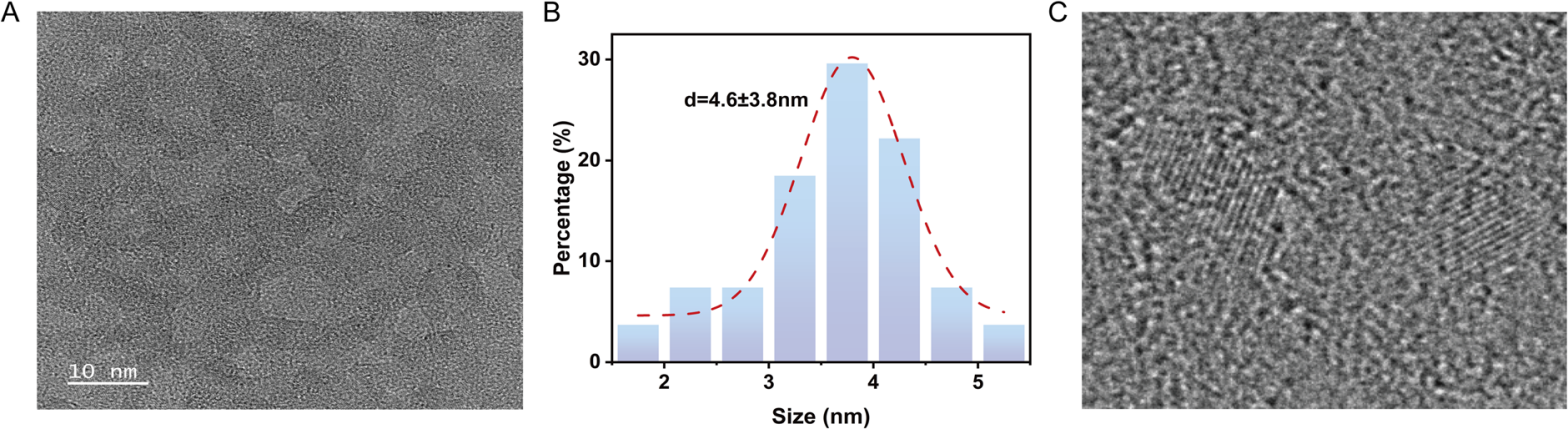
(A) A HRTEM image of N-CQDs. (B) The size distribution histogram of N-CQDs. (C) A partial enlarged view of the image in (A).

Fig. 1A and C show TEM imaging of N-CQDs, where clear lattice fringes can be observed. The particles are approximately spherical in shape and exhibit good dispersion. The corresponding particle size distribution statistics (Fig. 1B) indicate that the N-CQDs have a size distribution ranging from 0.8 to 8.4 nm, with an average particle size of 4.6 nm.

**Fig. 2:**
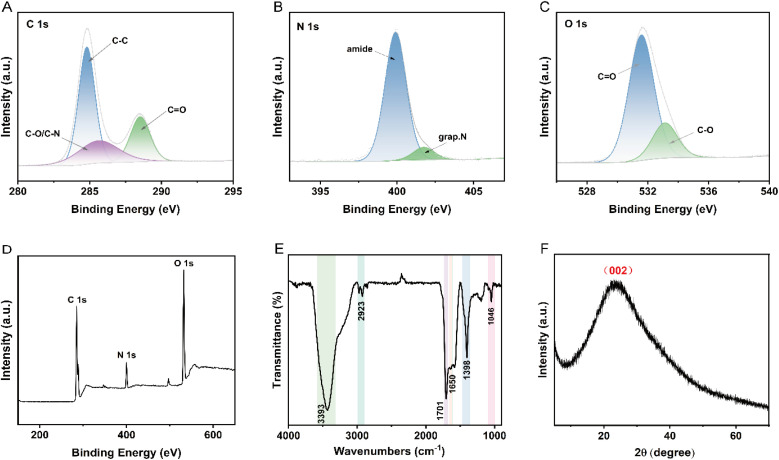
(A) The C 1s spectrum of N-CQDs. (B) The N 1s spectrum of N-CQDs. (C) The O 1s spectrum of N-CQDs. (D) The overall XPS spectrum of N-CQDs. (E) The FT-IR spectrum and (F) XRD pattern of N-CQDs.

XPS can reflect the elemental composition of a material. As shown in Fig. 2D, the sample has three typical peaks at 288, 400 and 532 eV, corresponding to C 1s, N 1s and O 1s, respectively. The elemental composition is 62.17% for C 1s, 8.81% for N 1s, and 29.02% for O 1s. Fig. 2A–C shows the high-resolution XPS spectra of N-CQDs: the C 1s spectrum can be fitted into three binding energy peaks, namely O=C (288.4 eV), C–O/C–N (285.6 eV) and C–C (284.8 eV);^32^ the O 1s spectrum shows two peaks at 531.6 and 533.2 eV, corresponding to C–O and C=O; and the N 1s spectrum exhibits two peaks at 401.3 eV and 399.9 eV, representing graphitic N and amide N.^33^

The presence of functional groups was confirmed by Fourier-transform infrared (FT-IR) spectroscopy. Fig. 2E shows the FT-IR characterization of N-CQDs: the surfaces of these N-CQDs all possess hydrophilic groups such as O–H/N–H (3393 cm^−1^),^34^ C–O (1046 cm^−1^),^35^ and C=O (1701 cm^−1^), ensuring their good solubility in aqueous solutions.^36^ The peak at 2923 cm^−1^ is assigned to the C–H stretching vibration. Strong absorption bands of the amide acid group are observed at 1701 and 1650 cm^−1^. The absorption band at 1701 cm^−1^ is attributed to C=O of the –COOH group of the amide acid, while the absorption band at 1650 cm^−1^ may be attributed to the secondary amino group of the amide acid group.^37^ The peak at 1398 cm^−1^ is assigned to the typical stretching vibration of C–N.^38^ The N-CQDs produced here contain characteristic functional groups such as –COOH and –OH, and all the data indicate consistency with the XPS results.^39^

**Fig. 3:**
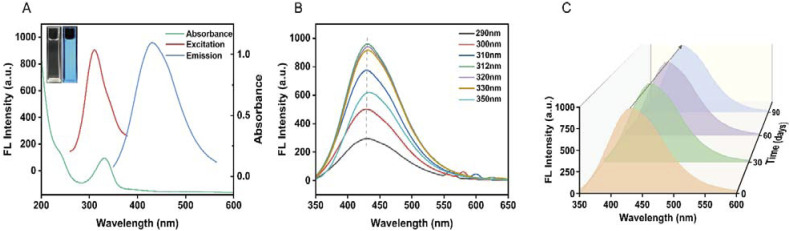
(A) The fluorescence spectrum and ultraviolet absorption spectrum of N-CQDs. (B) Emission spectra of N-CQDs at different excitation wavelengths. (C) Fluorescence spectra of N-CQDs after different storage times.

The optical properties of the N-CQDs were further investigated, including fluorescence spectra, UV-vis absorption spectra, and quantum yield. As shown in Fig. 3A, when the excitation wavelength was 312 nm, the maximum emission wavelength of the obtained N-CQDs in aqueous solution was 430 nm. The UV-vis absorption spectrum of N-CQDs showed a clear peak at 330 nm, which was related to its strong emission. The strong emission might be caused by the capture of excited-state energy by the surface state.^40^ The solution appeared as a transparent, slightly yellow liquid under visible light and emitted bright blue fluorescence when illuminated under a 365-nm UV lamp. Meanwhile, using quinine sulfate as a reference, the quantum yield of the N-CQDs was determined to be 5.6%. By monitoring the fluorescence intensity of the material after different storage times and measuring its fluorescence spectra, the change in fluorescence intensity over time was evaluated. The fluorescence intensity was measured every 30 days, and it was found that the fluorescence intensity remained essentially unchanged over 90 days (Fig. 3C), indicating that the N-CQD powder possesses good stability at room temperature.

### 3.2 Characterization of AgNPs

**Fig. 4:**
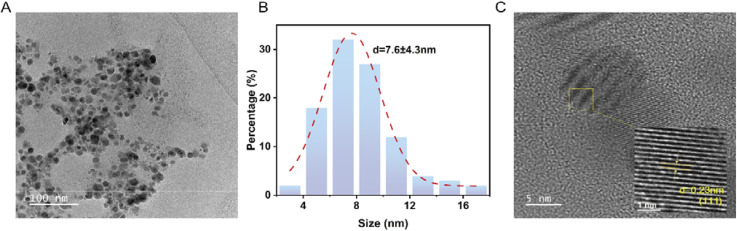
(A) A TEM image of AgNPs. (B) The size distribution histogram of AgNPs. (C) A HRTEM image of AgNPs.

Fig. 4A shows a transmission electron microscopy (TEM) image of AgNPs. The shape of AgNPs is nearly spherical, with an average particle size of 7.6 nm. High-resolution transmission electron microscopy (HRTEM) can reveal obvious lattice fringes, with lattice spacing of 0.23 nm (Fig. 4C), corresponding to the (111) crystal plane of Ag.^41^

**Fig. 5:**
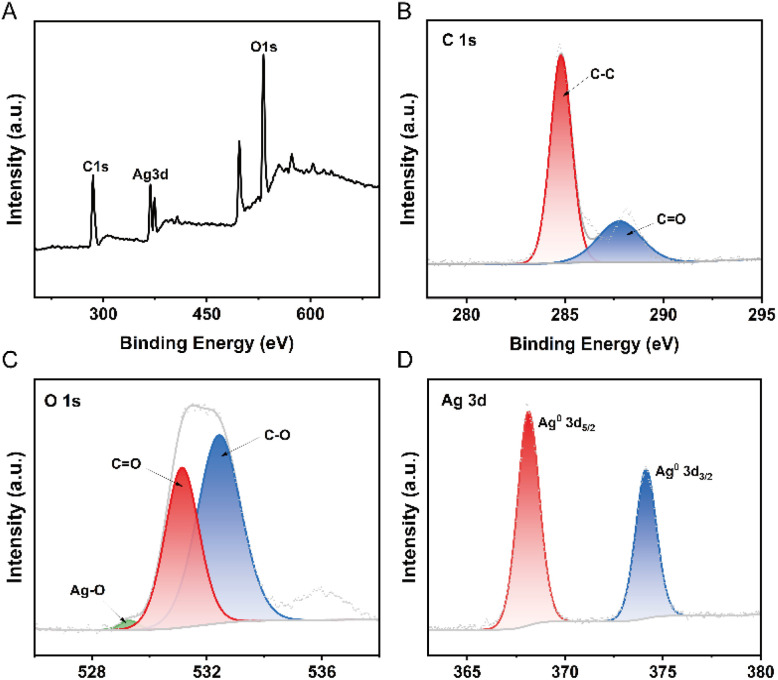
XPS spectra of AgNPs: (A) the overall spectrum; (B) the C 1s spectrum of AgNPs; (C) the O 1s spectrum of AgNPs; and (D) the Ag 3d spectrum of AgNPs.

In the XPS analysis of silver nanoparticles (Fig. 5D), the Ag 3d spectrum exhibits a doublet consisting of the Ag 3d_5/2_ and Ag 3d_3/2_ peaks; the binding energy of Ag 3d_5/2_ is approximately 368.2 eV. The binding energy of Ag 3d_3/2_ is approximately 374.2 eV, indicating that silver mainly exists in a metallic state (Ag^0^).^42^

### 3.3 Optimization of the detection conditions

For the concentration selection of N-CQDs, within the range of 0.02 to 0.2 mg mL^−1^, the fluorescence intensity of N-CQDs showed a trend of first increasing and then decreasing. When the concentration reached 0.1 mg mL^−1^, the fluorescence intensity achieved its maximum value. After exceeding this concentration, the fluorescence intensity gradually decreased. Therefore, 0.1 mg mL^−1^ was chosen as the optimal concentration of N-CQDs and was used for all subsequent experiments.

**Fig. 6:**
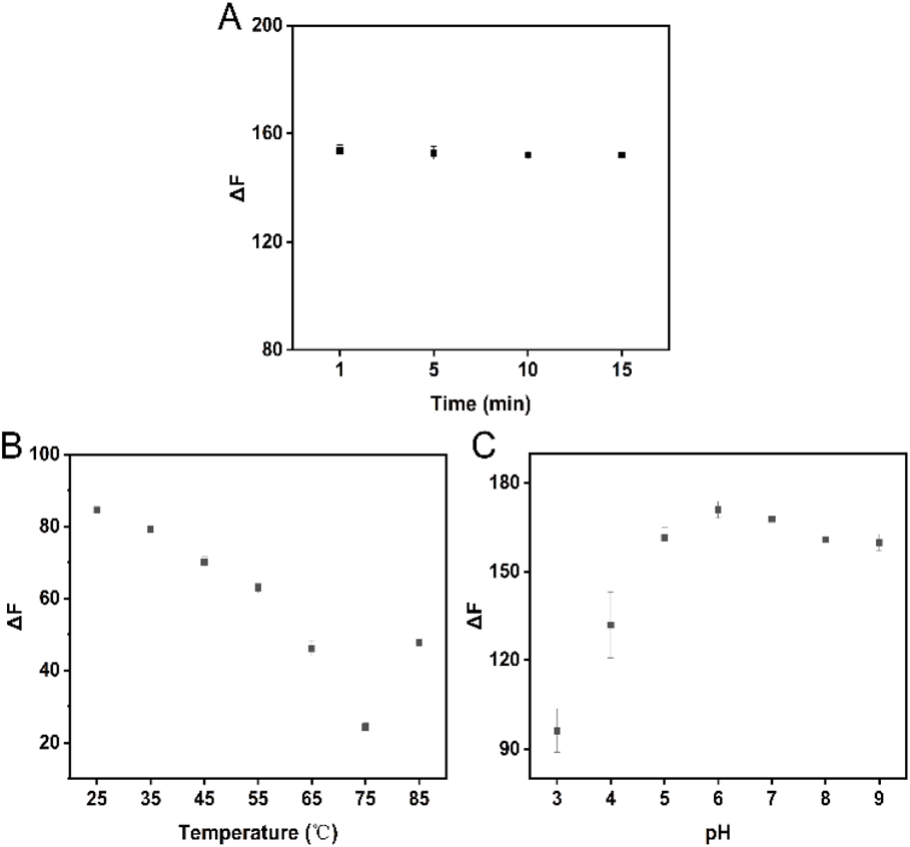
(A) The effect of time on the detection of thiram. (B) The effect of temperature on the detection of thiram. (C) The effect of pH on the detection of thiram.

To achieve the optimal detection performance for thiram, single-factor experiments were conducted to evaluate the impacts of pH, reaction temperature, and reaction time on the reaction system. By monitoring the fluorescence intensity within a time interval of 0–15 minutes, the impact of reaction time on the sensing performance was evaluated. As the fluorescence intensity remained unchanged with increasing time, it was inferred that the reaction was instantaneous, meaning that equilibrium was attained immediately after mixing. Therefore, a reaction time of 1 min was selected for the experiments (Fig. 6A).

To determine the optimal pH value, we observed the fluorescence response values within the pH range of 3 to 9 (Fig. 6C). At pH = 6, we found that the fluorescence response value reached its maximum, thereby confirming that this was the optimal condition for the reaction. The optimization of the reaction temperature was performed by observing the fluorescence response value within the range of 25 °C to 85 °C. The fluorescence response value was found to be maximum at 25 °C (Fig. 6B). The final reaction conditions were determined as follows: N-CQD concentration of 0.1 mg mL^−1^, reaction temperature of 25 °C, pH = 6, and reaction time of 1 minute. Under these conditions, the establishment of the standard curve was carried out.

### 3.4 Methodological investigation

**Fig. 7:**
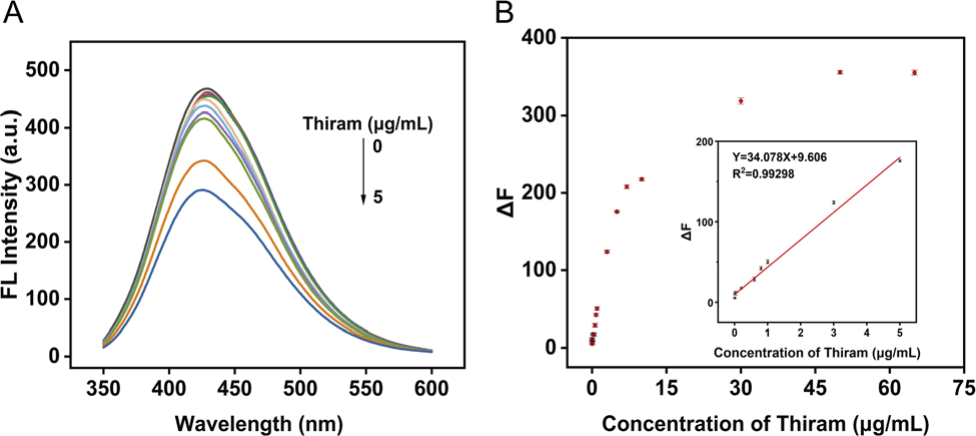
(A) Fluorescence curves of N-CQDs upon the addition of thiram at different concentrations. (B) The fitted calibration curve of the fluorescence change value (Δ*F*) *versus* thiram concentration.

Under the optimal conditions (N-CQD concentration: 0.1 mg mL^−1^, pH: 6, time: 12 min, temperature: 25 °C), the detection of thiram was performed. As shown in Fig. 7A, with the increase in thiram concentration, the fluorescence intensity of N-CQDs at 430 nm gradually decreased. A standard curve was established by plotting the fluorescence response value (Δ*F*, defined as *F*_0_ − *F*, where *F*_0_ and *F* represent the fluorescence intensity in the absence and presence of thiram, respectively) against the thiram concentration. Within the concentration range of 0.007 to 5 µg mL^−1^, a good linear relationship was observed between Δ*F* and the thiram concentration. The regression equation was Δ*F* = 34.078*x* + 9.61, with a correlation coefficient (*R*^2^) of 0.99298 (Fig. 7B). The theoretical detection limit (LOD) was calculated to be 0.002 µg mL^−1^.

### 3.5 Selectivity experiments

To evaluate its anti-interference ability, we investigated the effects of common environmental ions (Na^+^, Cu^2+^, Fe^2+^, SO_4_^2−^, and CO_3_^2−^) and pesticides (methyl parathion, glyphosate, and acephate). After the reaction under the optimal conditions, the fluorescence intensity change (Δ*F*) was measured at the optimal excitation wavelength. The N-CQDs/AgNPs sensing system exhibited a strong response to thiram (Fig. S1), while showing weak signals toward other pesticides and ions, indicating its excellent selectivity for thiram.

In addition, fungicides with a structure similar to that of thiram (ziram and disulfiram) were selected for testing under the same conditions. The results showed that the system had fluorescence responses to all three fungicides (Fig. S2). However, reproducibility analysis found that the system exhibited excellent detection stability for thiram, with small fluctuations in fluorescence response values in multiple parallel measurements. Although disulfiram and ziram could cause significant fluorescence changes in single tests, their repeatability was poor, with large fluctuations in fluorescence signals, making it difficult to obtain stable and reliable quantitative results. This also demonstrated the good selectivity of this method for thiram.

### 3.6 Analysis of actual samples

To further verify the practicality of the N-CQDs/AgNPs fluorescence sensing system, local soil samples and widely cultivated pears were selected for actual sample detection. Different concentrations of the thiram pesticide were added to these samples for spiked recovery experiments. In soil, the recovery rates of thiram ranged from 90.42% to 103.85%, and in pears, they ranged from 94.783% to 105.64%. These results indicate that this fluorescence sensor detection method possesses high accuracy and reliability, enabling the detection of thiram in complex samples (Table S1).

### 3.7 Study of the quenching mechanism

Fluorescence quenching mechanisms mainly include the dynamic quenching effect, the static quenching effect (SQE), fluorescence resonance energy transfer (FRET), the inner filter effect (IFE), and photoinduced electron transfer (PET).^43^

**Fig. 8:**
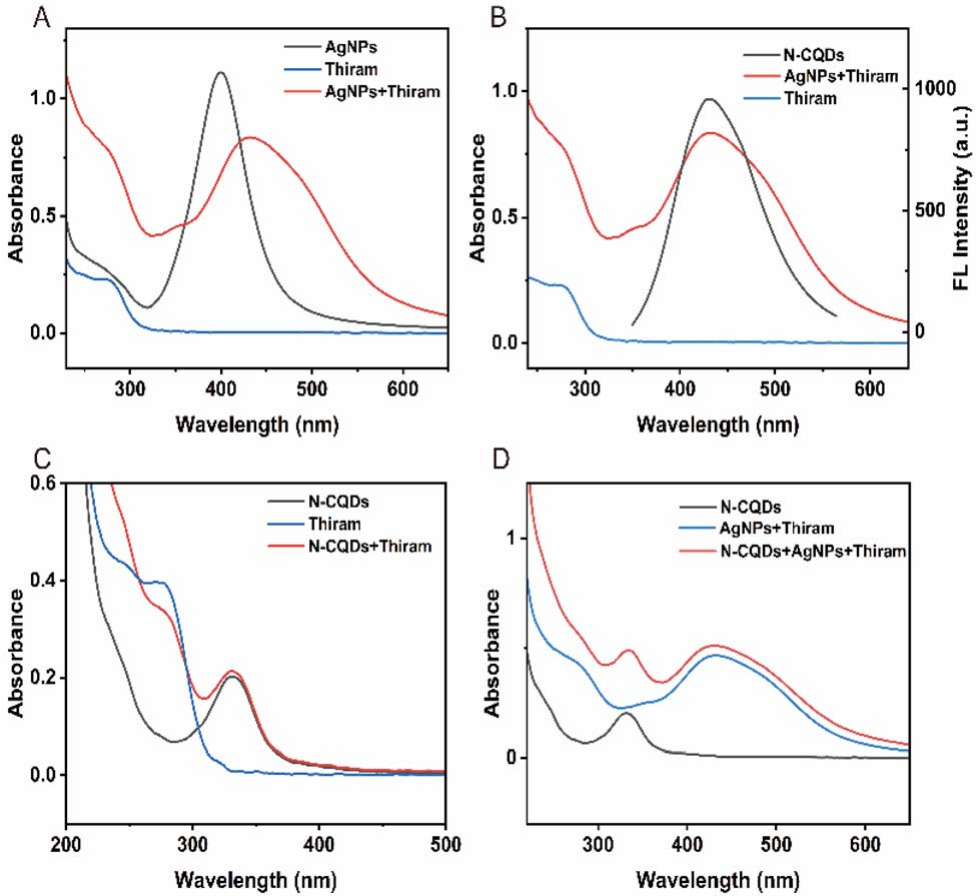
(A) Absorption spectra of AgNPs and thiram; (B) the emission spectrum of N-CQDs and absorption spectra of AgNPs-thiram and thiram; (C) absorption spectra of N-CQDs and thiram; and (D) absorption spectra of N-CQDs and AgNPs-thiram.

In this study, AgNPs were pre-mixed with thiram before being added to N-CQDs for detection. Therefore, the UV-vis absorption spectra of AgNPs before and after the addition of thiram were first compared (Fig. 8A) to investigate the reaction mechanism between AgNPs and thiram. As shown in Fig. 8A, the absorption spectrum of AgNPs with thiram revealed a redshift in the UV absorption peak and a change in the characteristic peak after thiram was added. The ultraviolet absorption spectrum after redshifting completely overlaps with the fluorescence emission spectrum of nitrogen-doped carbon quantum dots (Fig. 8B).

In order to rule out a direct quenching effect of thiram on nitrogen-doped carbon quantum dots, the UV absorption spectra of N-CQDs and thiram were compared (Fig. 8C), revealing that the spectrum of the mixture was merely a simple superposition of the individual spectra. No new characteristic peaks appeared in the absorption spectrum, nor did any of the original characteristic peaks disappear, indicating that no ground-state complex was formed and that there was no interaction between them. As shown in Fig. 8B, the fluorescence emission spectrum of N-CQDs did not overlap with the UV absorption spectrum of thiram. Moreover, thiram showed no significant absorption at the excitation and emission wavelengths of N-CQDs, indicating that there was no FRET or IFE. By comparing the fluorescence lifetimes of N-CQDs before and after the addition of thiram, it was found that the average fluorescence lifetime did not change significantly. Based on the above conditions, it can be concluded that thiram alone has no direct or indirect effect on the fluorescence properties of N-CQDs. This fluorescence sensing system enables the detection of thiram only in the presence of AgNPs.

By comparing the absorption spectra of N-CQDs before and after the addition of AgNPs-thiram (Fig. 8D), it was observed that the spectra merely exhibited simple superposition, with no new characteristic peaks appearing or existing peaks disappearing in the absorption spectra. This rules out the possibility of static quenching. As shown in Fig. 8B, the fluorescence emission spectrum of N-CQDs overlaps with the UV-vis absorption spectrum of AgNPs-thiram. Therefore, it is speculated that the quenching mechanism of N-CQDs may originate from FRET or the IFE. To further verify the mechanism, the fluorescence lifetimes of N-CQDs before and after the addition of AgNPs-thiram were measured (Fig. S3). Based on the amplitude-weighted average lifetime formula (eqn (2)), the fitting results of the fluorescence decay curves are shown in Table S2. The average fluorescence lifetime of N-CQDs did not change significantly upon the addition of thiram. Given that both FRET and dynamic quenching lead to a shortened fluorescence lifetime, the above results strongly rule out these two mechanisms as the main pathways. As shown in the data, the fluorescence lifetime of N-CQDs remained almost unchanged, indicating that the quenching of N-CQDs is not due to FRET but to the IFE.2
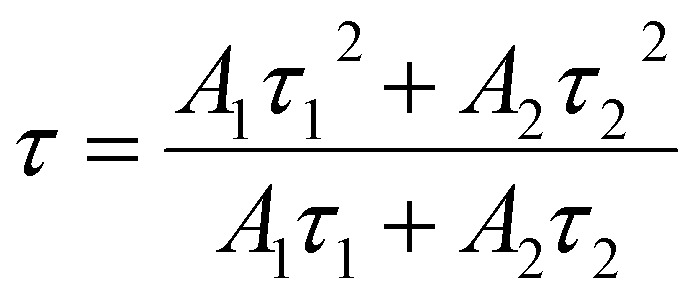


### 3.8 Comparison of detection methods for thiram

Table S3 (ref. 44–49) summarizes a systematic comparison between the N-CQDs/AgNPs fluorescence sensing system and other reported detection methods for thiram. Although each method has its own advantages in terms of the linear range or operational simplicity, the sensor proposed in this work achieves rapid detection and a lower detection limit (LOD). More importantly, it integrates several key advantages, including high sensitivity, a short detection time, and environmental friendliness.

## 4. Conclusions

In this study, nitrogen-doped carbon quantum dots (N-CQDs) with good water solubility and high stability were obtained through a one-step hydrothermal method. A fluorescence sensing system based on the inner filter effect (IFE) was constructed, which can be used for the highly sensitive detection of thiram, and it exhibits significant anti-interference ability against common ions and pesticides. The experimental results showed that the detection range of this system for thiram was 0.007–5 µg mL^−1^, with an excellent linear response and a limit of detection (LOD) as low as 0.002 µg mL^−1^. This method was applied to the detection of real soil and pear samples, with recovery rates ranging from 94.783% to 105.64% and relative standard deviation (RSD) values of 0.71–5.62%. It demonstrated high accuracy and sensitivity. Furthermore, the N-CQD powder exhibited good stability at room temperature, further verifying its potential for practical applications. This method provides a new strategy for the rapid and simple detection of thiram.

## Author contributions

Shangyu Hou: writing – original draft. Rong Yang: data curation, conceptualization. Pute Yang: data curation, methodology. Xiaoqian Liu: formal analysis. Yu Gu: formal analysis, data curation. Qin Zhou: writing – review and editing, supervision.

## Conflicts of interest

There are no conflicts to declare.

## Supplementary Material

RA-OLF-D6RA04161B-s001

## Data Availability

Data will be made available on request. Supplementary information (SI): selectivity experiments against other pesticides, cations, anions, and structurally similar pesticides; fitted fluorescence lifetime decay curves of N-CQDs with various substances; spike recovery tests in real samples; fluorescence lifetimes of N-CQDs under different conditions; and a comparison with other thiram detection methods. See DOI: https://doi.org/10.1039/d6ra04161b.

## References

[cit1] Wang C., Zhu Z., Huang X., Wang X., Zhang L., Peng Y., Wan R., Han L., Li L., Qin X., Li H., Chen J. (2024). J. Agric. Food Chem..

[cit2] Liu K., Li Y., Iqbal M., Tang Z., Zhang H. (2022). Chemosphere.

[cit3] Charoenkitamorn K., Chailapakul O., Siangproh W. (2015). Talanta.

[cit4] da Silva R. C., Wickert C., Pizzutti I. R., de Kok A. (2021). J. Agric. Food Chem..

[cit5] Lehotay S. J., de Kok A., Hiemstra M., Van Bodegraven P. (2005). J. AOAC Int..

[cit6] Gueguen F., Boisdé F., Queffelec A. L., Haelters J. P., Thouvenot D., Corbel B., Nodet P. (2000). J. Agric. Food Chem..

[cit7] Li H., Hu Y., Lin Z., Yan X., Sun C., Yao D. (2024). Food Chem..

[cit8] Li W.-K., Shi Y.-P. (2024). TrAC, Trends Anal. Chem..

[cit9] Herrera-Chacon A., Ceto X., del Valle M. (2021). Anal. Bioanal. Chem..

[cit10] Yaqoob S. B., Adnan R., Rameez Khan R. M., Rashid M. (2020). Front. Chem..

[cit11] Sharma S. , Shekhar S., Gautam S., Sharma B., Kumar A. and Jain P., Carbon-based nanomaterials as novel nanosensors, in Nanofabrication for Smart Nanosensor Applications, Elsevier, 2020, pp. 323–347.

[cit12] Kong J., Wei Y., Zhou F., Shi L., Zhao S., Wan M., Zhang X. (2024). Molecules.

[cit13] Bakirhan N. K. and Ozkan S. A., Handbook of Nanomaterials for Industrial Applications, Elsevier, 2018, pp. 520–529.

[cit14] Mansuriya B. D., Altintas Z. (2021). Nanomaterials.

[cit15] Wang X., Sun G., Li N., Chen P. (2016). Chem. Soc. Rev..

[cit16] Zulkfly A. F., Iqbal A., Ida J., Mydin R. B. S. M. N., Noh N. A. M., Hussin M. H., Al-Fatesh A. S., Ibrahim A. A. (2024). Chem. Afr..

[cit17] Mohandoss S., Roy P., Ahmad N., Gomez P. L. A. M., Velu K. S., Somu P., Kim S.-C. (2026). Inorg. Chem. Commun..

[cit18] Yang H.-L., Bai L.-F., Geng Z.-R., Chen H., Xu L.-T., Xie Y.-C., Wang D.-J., Gu H.-W., Wang X.-M. (2023). Mater. Today Adv..

[cit19] Wu H., Yan Y., Peng Q., Tang Y. (2025). Appl. Phys. Rev..

[cit20] Liu Y., Jiang L., Li B., Fan X., Wang W., Liu P., Xu S., Luo X. (2019). J. Mater. Chem. B.

[cit21] Guo X., Xu L., Zhang L., Wang H., Wang X., Liu X., Yao J., Hao A. (2018). J. Lumin..

[cit22] Zhu R., Huang W., Ma X., Zhang Y., Yue C., Fang W., Hu Y., Wang J., Dang J., Zhao H., Li Z. (2019). Anal. Chim. Acta.

[cit23] Zhan Y., Geng T., Liu Y., Hu C., Zhang X., Lei B., Zhuang J., Wu X., Huang D., Xiao G., Zou B. (2018). ACS Appl. Mater. Interfaces.

[cit24] Ma Y., Wang R., Qin X., Zhang Q., Zhong X. (2025). Plasma Processes Polym..

[cit25] Trang T. T., Pham T. T. H., Dang N. V., Nga P. T., Linh M. V., Vu X. H. (2024). RSC Adv..

[cit26] Tantawy M. A., Farag M. A., Yehia A. M. (2020). New J. Chem..

[cit27] Gaviria-Arroyave M. I., Cano J. B., Peñuela G. A. (2020). Talanta Open.

[cit28] Moores A., Goettmann F. (2006). New J. Chem..

[cit29] Liu G., Kong D., Han J., Zhou R., Gao Y., Wu Z., Zhao L., Wang C., Wang L., Lu G. (2021). Sens. Actuators, B.

[cit30] Li Z., Wang Y., Ni Y., Kokot S. (2014). Sens. Actuators, B.

[cit31] Hasan H. A., Abdulmalek E., Rahman M. B. A., Shaari K. B., Yamin B. M., Chan K. W. (2018). Chem. Cent. J..

[cit32] Qu D., Zheng M., Zhang L., Zhao H., Xie Z., Jing X., Haddad R. E., Fan H., Sun Z. (2015). Sci. Rep..

[cit33] Hola K., Sudolska M., Kalytchuk S., Nachtigallova D., Rogach A. L., Otyepka M., Zboril R. (2017). ACS Nano.

[cit34] Jeyasubramanian K., Muthuselvi M., Hikku G. S., Muthusankar E. (2019). J. Colloid Interface Sci..

[cit35] Peng H., Travas-Sejdic J. (2009). Chem. Mater..

[cit36] Wang B., Cai H., Waterhouse G. I. N., Qu X., Yang B., Lu S. (2022). Small Sci..

[cit37] Guo Y., Zhao W. (2020). Spectrochim. Acta, Part A.

[cit38] Jain P., Choudhary V., Varma I. K. (2003). Eur. Polym. J..

[cit39] Gao R., Wu Z., Wang L., Liu J., Deng Y., Xiao Z., Fang J., Liang Y. (2020). Nanomaterials.

[cit40] Li J., Jiao Y., Feng L., Zhong Y., Zuo G., Xie A., Dong W. (2017). Microchim. Acta.

[cit41] Duan X., Chen T., Chen T., Huang L., Ye L., Lo B. T. W., Yuan Y., Edman Tsang S. C. (2021). Chem. Sci..

[cit42] Cui Y., Ma J., Gao G., Duan X., Ying M., Huang L., Li M. (2025). BMC Biotechnol..

[cit43] Long C., Jiang Z., Shangguan J., Qing T., Zhang P., Feng B. (2021). Chem. Eng. J..

[cit44] Zhao X., Kong D., Jin R., Li H., Yan X., Liu F., Sun P., Gao Y., Lu G. (2019). Sens. Actuators, B.

[cit45] Walia S., Sharma R. K., Parmar B. S. (2009). Bull. Environ. Contam. Toxicol..

[cit46] Mei H., Shu H., Lv M., Liu W., Wang X. (2020). Microchim. Acta.

[cit47] Malik A. K., Faubel W. (2000). Anal. Lett..

[cit48] Zhang Y., Zhao J., Sun X., Pan W., Yu G., Wang J. (2018). Sens. Actuators, B.

[cit49] Yang Z., Hu L., Ning K., Wu Y., Liang J. (2023). Food Chem..

