# Supplementary material for: Construction of a nitrogen-doped carbon quantum dot-silver nanoparticle composite fluorescence sensing system for the highly selective detection of thiram in soil and pears
Source: RSC Adv. 2026 Jul 15. Online ahead of print. doi: 10.1039/d6ra04161b (PMC13370907; doi:10.1039/d6ra04161b)
Supplement: RA-OLF-D6RA04161B-s001 [file RA-OLF-D6RA04161B-s001.pdf]

Supplementary Information (SI) for RSC Advances.
This journal is © The Royal Society of Chemistry 2026

Supplementary materials

Rapid Detection of Thiram Based on Nitrogen-Doped Carbon Quantum Dots-Silver Nanoparticles Fluorescence Sensing System

Shangyu Hou [a],Rong Yang [1,a],Pute Yang [a],Xiaoqian Liu [a], Yu Gu [a], Qin Zhou [a,b]*

([a]College of advanced agriculture and ecological environment, Heilongjiang university, Harbin, 150080;

[b]Inspection and Test Center of Beet Quality, Ministry of Agriculture and Rural Afairs, Harbin 150080, China)

* Correspondence author.
E-mail address: zhouqin@hlju.edu.cn (Qin Zhou)
[1]These authors contributed equally to this work.

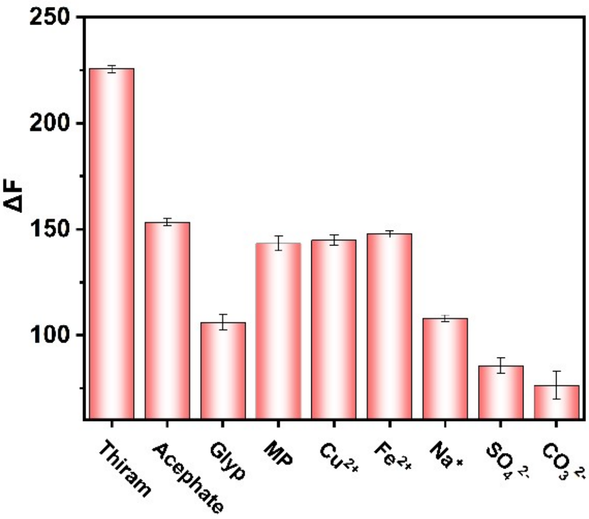

Figure S1 Comparison of fluorescence responses between thiram and interfering substances

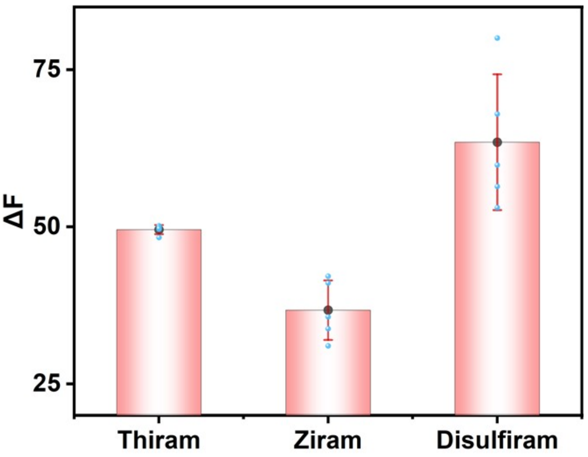

Figure S2 Comparison of fluorescence responses between thiram and structurally similar substances

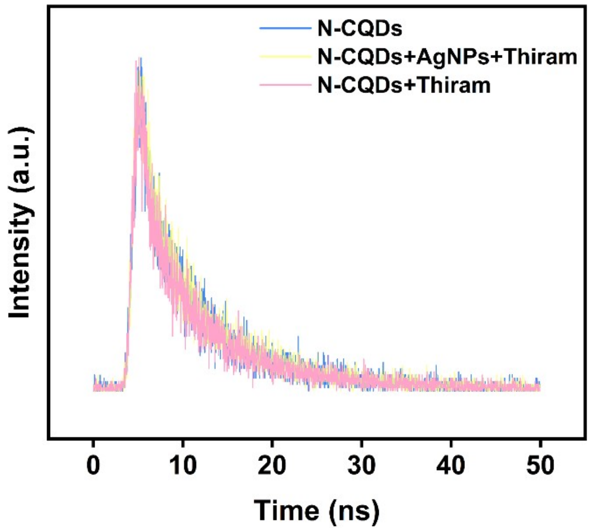

Figure S3 Fluorescence decay curves of N-CQDs in the presence of different substances

Table S1. Results of the spiked recovery experiment for thiram in actual samples

Sample	Spiked (mg/L)	F (a.u.)	ΔF (a.u.)	Detected (mg/L)	RSD (%)	Recovery rate(%)
Soil	Blank	315.38	/	/	1.05	/
	0.6	287.75	29.627	0.59	4.56	97.896
	1	273.76	41.617	0.94	2.70	93.921
	3	213.33	102.05	2.71	3.15	90.421
	5	128.81	186.57	5.19	2.04	103.85
Pear	Blank	372.89	/	/	0.81	/
	0.6	343.56	29.335	0.58	5.62	96.454
	1	330.57	42.324	0.96	3.66	95.996
	3	255.28	117.61	3.17	2.66	105.64
	5	201.78	171.11	4.74	0.71	94.783

Table S2. The fluorescence lifetime of N-CQDs in the presence of different substances.

Substance	τ_1 (ns)	τ_2 (ns)	A_1	A_2	χ^2	Average Lifetime (ns)
N-CQDs	1.17	7.20	33.76	54.21	0.95	6.64
N-CQDs+ AgNPs-Thiram	1.46	7.16	34.79	60.80	0.92	6.56
N-CQDs+ Thiram	1.25	7.00	37.43	54.21	0.98	6.37

Table S3. Comparison between this study and other reported methods for thiram detection.

Method	Linear Range	LOD	Time(min)	Reference
Fluorescence method	0.05-0.5 μg/mL	0.025 μg/mL	2	44
HPLC	2-100 μg/mL	0.2 μg/mL	20	45
Fluorescence method	33-670 nM	9.90 nM	15	46
Capillary electrophoresis method	0.5-240 μg/mL	0.5 μg/mL	6	47
Fluorescence method	0-12 μg/mL	0.75μg/mL	180	48
Fluorescence method	0.012-2 μM	0.01 μM	10	49
Fluorescence method	0.007-5 μg/mL	0.002 μg/mL	1	This work
